# Mental health treatment outcomes in a humanitarian emergency: a pilot model for the integration of mental health into primary care in Habilla, Darfur

**DOI:** 10.1186/1752-4458-3-17

**Published:** 2009-07-21

**Authors:** Renato Souza, Silvia Yasuda, Susanna Cristofani

**Affiliations:** 1 Médecins Sans Frontières – Operational Center, Geneva, Rue de Lausanne 78. CH-1211 Geneva, Switzerland; 2 UNIFESP. Federal University of Sao Paulo. Rua Botucatu, 740. 4° Andar. Sao Paulo, Brazil

## Abstract

**Background:**

There is no description of outcomes for patients receiving treatment for mental illnesses in humanitarian emergencies.

MSF has developed a model for integration of mental health into primary care in a humanitarian emergency setting based on the capacity of community health workers, clinical officers and health counsellors under the supervision of a psychiatrist trainer.

Our study aims to describe the characteristics of patients first attending mental health services and their outcomes on functionality after treatment.

**Methods:**

A total of 114 patients received mental health care and 81 adult patients were evaluated with a simplified functionality assessment instrument at baseline, one month and 3 months after initiation of treatment.

**Results:**

Most patients were diagnosed with epilepsy (47%) and psychosis (31%) and had never received treatment. In terms of follow up, 58% came for consultations at 1 month and 48% at 3 months.

When comparing disability levels at baseline versus 1 month, mean disability score decreased from 9.1 (95%CI 8.1–10.2) to 7.1 (95%CI 5.9–8.2) p = 0.0001.

At 1 month versus 3 months, mean score further decreased to 5.8 (95%CI 4.6–7.0) p < 0.0001.

**Conclusion:**

The findings suggest that there is potential to integrate mental health into primary care in humanitarian emergency contexts. Patients with severe mental illness and epilepsy are in particular need of mental health care.

Different strategies for integration of mental health into primary care in humanitarian emergency settings need to be compared in terms of simplicity and feasibility.

## Background

Despite the fact that mental disorders are commonly encountered among populations facing humanitarian emergencies, little research has been done on the description of treatment models for the integration of mental health care into primary health care in the midst of a humanitarian emergency and their outcomes. Most of the research has focused on posttraumatic stress disorder and little attention has been given to people suffering with severe mental disorders [1,2]. Added to that, most of the studies do not assess the effect of interventions on daily functioning, an outcome variable of fundamental importance for populations facing the extreme adversities of humanitarian emergencies [1,3].

Médecins Sans Frontières (MSF), an international non-governmental organisation, has implemented primary health care activities in Habilla, Darfur since 2004. MSF started to develop Mental Health activities in Habilla in accordance with the recommendations of the health section of the Inter Agency Standing Committee (IASC) guidelines on "Mental Health and psychosocial support in emergency settings" [4]. The MSF program aimed to develop a pilot model for the integration of mental health into medical care activities in a humanitarian emergency. The process of integration started in April 2007. Treatment of mental illnesses and epilepsy had never been available to the population in this area of West Darfur.

The objective of the present study is to evaluate our pilot model of integration of mental health into primary health care activities in a humanitarian emergency by focusing on: (1) the description of the population first attending the mental health services and by (2) determining whether this pilot intervention improves functionality of adults under mental health care.

## Methods

### Setting

Violence in Darfur began in 2003 when rebel groups complaining of discrimination against black Africans began attacking government targets.

The government mobilised what it called "self-defence militias" in response; but denies any links to the Janjaweed which is accused of trying to "cleanse" black Africans from Darfur.

According to the United Nations (UN); in 2007, 270,000 people were displaced adding to the already 2 million that have been displaced since the beginning of the conflict in Darfur [5].

Habilla, in West Darfur, a city of 30,000 has increased in size with the addition of approximately 20,000 internally displaced persons (IDP). It is an area of limited humanitarian access, only accessible by UN helicopter when security permits.

A rapid health assessment conducted by MSF between the 29^th ^June and 10^th ^July of 2005 described a crude mortality rate of 0.4/10 000/day in a retrospective recall period of 181 days and a under 5-mortality rate of 0.5/10 000/day. The main causes of death being watery diarrhoea and fever with shivering. (MSF nutritional survey and rapid health assessment – internal document)

Only two health structures provided medical care in Habilla. MSF health care center provided out-patient department (OPD) services with an estimation of 30,000 consultations per year and an in-patient department with on average 500 admissions per year. Reproductive health care services, a therapeutic feeding program and treatment for tuberculosis were also available. The health centre was open everyday of the week for medical emergencies and OPD consultations were performed by one medical doctor and three clinical officers five days per week. Twenty two community health workers were responsible for establishing the link between the community and the medical services provided by MSF.

### Procedures

We used mental health assessment instruments in routine consultations from April to November 2007.

The sampling framework consisted of the first 114 consecutive patients that attended our services during the initial six months of project implementation. The period of 6 months was chosen as this was the timeframe we had established to perform the first analysis of data and according to results plan the future directions of the project.

### Instruments

The assessment instruments were administered by the health counsellor and consisted of a questionnaire divided into 4 parts:

a. socio-demographic items,

b. a list of 5 syndromic mental health diagnoses (common mental disorders (CMD), psychosis, alcohol and substance abuse or dependence, epilepsy and children's mental health problems),

c. a list of 25 symptoms of depression and anxiety from the Hopkins symptoms checklist (HSC) [6]

d. 3 questions on adult functionality adapted from the Medical Outcome Studies 36-Item Short Form Health Survey questionnaire (SF-36) [7].

### Questionnaire development

In order to ensure cultural appropriateness and linguistic accuracy, bilingual staff including a local doctor with previous mental health training and the psychiatrist-trainer reviewed contextual meaning of all questions. Revisions were made where needed after piloting the questionnaire.

In order to have a simple instrument to evaluate changes in functionality, our main outcome, we developed a 3 item-scale by adapting questions from the SF-36. The SF-36 is a self-report or interviewer administered measure of health related quality of life. It is probably the best validated and most widely used measure of global health and disablement internationally [8].

The 3 questions, aimed to reflect areas of functionality of the local population. The questions were:

1. How is your health?

2. To what extend does your problem interfere with your normal social activities?

3. How much of the time your problem interferes with your social activities?

These questions were followed by an explanation about what was meant by health using local concepts. Detailed explanations about social activities appropriate for the gender, age and position of the person in the society, and time perceptions in the local community were also given with examples (e.g. daily activities such as cooking; cleaning; care for the family and children; interaction with the community, hygiene, personal and traditional duties and work).

Responses were given on a Likert scale (0 to 4) with higher scores reflecting higher levels of disability.

Grading of responses involved an interactive discussion about patient's functionality.

The same national staff counsellor was responsible for all interviews. All questions referred to the last 7 days.

A total disability score was derived ranging from 0 to 12

### Psychometric properties of the 3 items disability scale

For convergent validity, we correlated the total score of the 3 item disability scale against the total score of the HSC and the total score on number of traumatic events. We expected the disability score to correlate more strongly with the HSC than with the trauma score.

We found a correlation of 0.60 (p < 0.001) with the HSC and no evidence of correlation with trauma.

For a measure of "known group validity", we tested the hypothesis that different diagnostic groups have different disability scores. ANOVA results demonstrated strong evidence that the group with psychosis had the highest mean score on disability followed by CMD and epilepsy.

The scale was highly internally consistent with a Cronbach's alpha of 0.95 (Table 1).

**Table 1:** Psychometric properties of the 3 items disability scale

**Convergent validity measure**		
	**HSC score (N = 46)**	**Trauma score (N = 63)**

	r	r
**Disability Scale score**	0.60	-0.07
	p < 0.001	p = 0.60

**Known group validity measure (N = 69)**		

	**Mean Disability score (SD)**	**Frequency**

**CMD**	7.80 (4.76)	5
**Epilepsy**	6.34 (3.40)	29
**Psychosis**	10.83 (2.16)	35
**ANOVA p < 0.001**		

**Internal consistency (N = 81)**		

	**Cronbach's alpha**	

**Disability Scale**	0.95	

### Intervention

#### A. Program development

In order to start the integration of mental health into primary care we added the following resources to the existing medical program:

a. Psychiatrist-trainer

b. Basic psychiatric drug package

c. One extra health counsellor for out patient department (OPD)

d. MSF Mental Health treatment guidelines.

A small library with essential mental health books and training videos were also incorporated into the existing medical library of the medical program.

Although our initial assessment showed that mental health care was never available to the population of Habilla, key mental health conditions could be identified by understanding the local idiom of distress. (Table 2) We learned that treatment of mental health conditions was under the domain of traditional or religious practices that the population had judged ineffective. Based on the results of our assessments, we decided to concentrate our case finding strategies at different levels.

**Table 2:** Local idiom of distress in Habilla, Darfur

**Nafsyiat**: the person becomes persistently sad and isolated, crying frequently and refusing to eat. Suicidal ideas can happen. It can happen after bad events or trauma. People with nafsyiat can disconnect from the reality and become "mad". It probably corresponds to severe depression.	**Magnun**: the person is completely disconnected from the reality, being aggressive, hearing voices and speaking to herself. It is considered a permanent possession by evil spirits and no local treatment is able to help this person. When disruptive, they are tied to trees or posts. It probably corresponds to different forms of psychosis.	**Sahra**: episodic loss of conscience with movements of arms and legs. It is considered an episodic possession by evil spirits. It probably corresponds to tonic-clonic generalized seizures and conversive disorders.

We developed the program by focusing on the training of health care professionals to be able to identify cases of CMD, alcohol and substance abuse and dependence at the outpatient level and psychosis, epilepsy and children with mental health problems at the community level. Case definitions were developed for each of the 5 conditions.

We did not develop any special facility for assessment and treatment of patients with mental disorders. We aimed to combat any stigma towards these conditions by diagnosing and treating them in the same setting as any other medical condition that was part of the primary health care package.

All treatment was provided free of charge and when medication was prescribed, it was supplied until the next consultation up to the end of the treatment.

Despite the fact that Habilla was classified as a conflict zone, during the time where MSF activities were in place, the situation remained stable. Nevertheless, as a precautionary measure, we planned strategies to avoid program disruption in order to minimize the risk that patients would have to interrupt their psychopharmacological treatment due to possible deterioration of the security situation.

#### B. Human resources and training

The integration of mental health care consisted of the training of personnel working at 3 different levels:

1. Community Health Workers: Responsible for sensitisation of the community about the main symptoms of 5 syndromic mental illnesses (CMD, psychosis, alcohol and substance abuse or dependence, epilepsy and children's mental health problems) and the availability of treatment at health care level. Active case finding and referral of cases of psychosis, epilepsy and children with mental health problems to the health centre was considered their core responsibility. Community health workers performed patient outreach for adherence purposes.

2. Medical staff: Responsible for the clinical diagnosis of the five mental health conditions and their psychopharmacological treatment, including side-effect management and evaluation of treatment response. Active case finding for patients with CMD, alcohol, and drug abuse problems was conducted at the outpatient department level.

3. Health Counsellor: Responsible for providing patient and family mental health therapeutic education to all patients. Mental Health Counselling was offered as a private consultation with the health counsellor helping patients and families to solve their problems related to each specific mental health condition.

Mental Health treatment guidelines were developed for the 5 syndromic mental illnesses based on WHO recommendations and national essential drug list [9]. Mental health counselling and psychopharmacological treatment guidance was simplified via the use of flow-charts and tables guiding medical staff on how to use these drugs.

#### C. Clinical care

In order to diagnose the 5 mental health syndromes, we used diagnostic criteria and flow charts based on a problem-based approach developed by Vikram Patel in "Where there is no psychiatrist" [10].

The choice of psychopharmacological treatment was based on national availability of psychotropic drugs.

Treatment guidelines consisted of:

Epilepsy: phenobarbital up to 120 mg or carbamazepine up to 1000 mg for a minimum of one year.

Psychosis: chlorpromazine up to 400 mg or haloperidol up to 10 mg and biperidene as an anticholinergic for a minimum of 6 months.

CMD: amytriptiline up to 150 mg for severe depression for up to 6 months. Mild and moderate cases receive 8 sessions of problem-solving counselling.

Alcohol and substance abuse or dependence: diazepam up to 80 mg for alcohol withdrawal and 8 sessions of problem-solving counselling for the management of alcohol or substance abuse and dependence.

Children's mental health problems: 8 sessions of problem-solving counselling.

### Analysis

After interviews were completed, data were entered into a MS Excel database developed for program monitoring.

Paired t-test was used to assess differences in functionality for the first 81 consecutive adults attending mental health care elicited at baseline, 1 and 3 months. Defaulters (missing values at follow up) were excluded from the analysis.

Patient data was entered anonymously and all data collected by the health counsellor was done under the supervision of the psychiatrist trainer.

Analyses were conducted in Stata 9.2 [11].

### Ethical considerations

The Medecins Sans Frontieres Ethical Review Board granted permission for the authors to present the results of the program evaluation with the previously collected data. Study protocol and objectives were explained to participants in the local language prior to their inclusion and oral informed consent was obtained.

## Results

From April to November 2007, we received 114 patients, 81 were adults.

The proportion of males was similar to females, most of the patients were between 18 and 44 years old, the majority were single.

60% were internally displaced and 80% relied on humanitarian aid. Most had no education. (Table 3) The majority of patients had epilepsy and psychosis. (Table 4)

**Table 3:** Sociodemographic characteristics of patients with mental illness attending mental health consultations at Habilla Health Center.*

**Socio-demographic characteristics of patients in mental health care**		
	**Frequency**	**%**

**Gender**		
Male	58	50.9
Female	56	49.1
		
**Age (years)**		
0–17	33	28.9
18–44	62	54.4
45-	18	16.7
		
**Marital status (n = 109)**		
Single	74	67.8
Married	17	15.7
Divorced/widowed	18	16.5
		
**Origin (n = 110)**		
Displaced	66	60.0
Local	44	40.0
		
**Education (n = 109)**		
No education	91	83.5
Primary	16	14.9
Secondary	2	1.6
		
**Financial status (n = 110)**		
Family or self support	23	20.9
External aid	87	79.1

* N = 114 unless stated otherwise due to unavailability of information at first consultation.

**Table 4:** Distribution of patients according to 5 syndromic mental illness diagnosis (N = 114)

**Mental Health diagnosis at baseline**		
	**Frequency**	**%**

**Diagnosis**		
CMD	5	4.4
Alcohol and substance abuse or dependence	1	0.9
Epilepsy	54	47.4
Psychosis	35	30.7
Children's mental health problems	13	11.4
Others	6	5.3

For the evaluation of the main outcome, changes in functionality scores, all 81 adults had scores at baseline, 47 at 1 month (more or less 15 days) and 39 around 3 months.

We found strong evidence of decrease on disability score at 1 month and at 3 months when compared with baseline scores. From 1 month to 3 months, there was some evidence of a decrease in scores. (Table 5)

**Table 5:** Time changes in disability scores for adults followed on mental health care

**Changes in Disability score (N = 47)**		
**Variable**	**Mean**	**95% CI**

**Baseline**	9.13	8.08 – 10.19

**1 month**	7.09	5.92 – 8.25

P = 0.0001		
**Changes in disability score (N = 49)**		

**Variable**	**Mean**	**95% CI**

**Baseline**	9.33	8.16 – 10.50

**3 months**	5.77	4.57 – 6.96

P < 0.0001		
**Changes in disability score (N = 36)**		

**Variable**	**Mean**	**95% CI**

**1 months**	6.86	5.43 – 8.29

**3 months**	5.69	4.43 – 6.95

P = 0.09		

## Discussion

This pilot model, focusing on the diagnosis and treatment of 5 syndromic mental illnesses and completely integrated into existing primary health care services was feasible in Habilla.

Our findings seem to demonstrate that patients improve their functionality as is seen in non-war contexts.

To our knowledge, this is the first evaluation based on real life activities to demonstrate positive outcomes for patients treated for severe mental health problems in a humanitarian emergency.

As others, we also observed that after the initiation of mental health services in an area that had never had access to diagnosis and treatment of mental illnesses, the first patients to attend are patients with severe mental illness and epilepsy [2]. We believe that it is fundamental to address immediately the needs of these patients.

We also speculate that the low numbers of patients with CMD diagnosed by our services was due to the fact that in the first 6 months, we were unable to train properly the medical staff on the recognition and diagnosis of CMD. As a result, we plan to address this problem by developing and testing algorithms where CMD can be diagnosed in patients presenting at outpatient level with multiple unexplained somatic complaints as these are known to be strongly associated with depression and anxiety in developing countries [12].

We believe that the low numbers of cases of alcohol and drug abuse identified in our services was due to the religions restrictions on consumptions of these substances in Darfur.

We have decided not to measure outcomes for children identified with mental health problems due to the lack of availability of simple and short instruments that can be incorporated into mental health services in remote areas and humanitarian emergencies. Most of the cases labelled as child mental health problems identified by us were cases of intellectual disability. We are also aware that we have not properly developed clear case definitions for specific children's mental health problems that can be found in conflict situations (e.g. enuresis, post traumatic stress disorder). There is a clear need to identify children's mental health problems of public health importance in humanitarian emergencies in order to help organisations to prioritise the delivery of services for key mental health conditions.

We are of course worried with levels of defaulting and future research should investigate possible determinants of defaulting in mental health services in humanitarian emergencies. We speculate that defaulting was associated with poor family support and distance from the health centre. Unfortunately, we have not included these variables in our monitoring system and therefore can not test these hypothesis here.

There is a strong need for organisations to share their strategies on how the integration of mental health into primary health care services can be better accomplished and especially in contexts like Darfur, or other emergencies.

We believe that this integration is highly context specific. Here, we struggled to treat patients that had never received any treatment before, staff needed considerable training and we are in an extremely violent area. In Beirut, in 2006, we received patients that were already on treatment but left tablets at home while escaping from aerial bombardments. In that setting, we struggled to keep them on treatment by having an essential list of drugs and relying on qualified local professionals. (MSF internal unpublished report)

This study has very important limitations. Being a small study as it is based on real life activities, caution is needed on the interpretation of the findings.

We acknowledge that having chosen only 5 syndromic diagnoses in order to integrate mental health into primary health care can be considered too simplistic. Our 5 syndromes approaches could also have missed cases of mental disorders that are expected to be seen in war contexts, such as acute stress disorder and post-traumatic stress disorder [13].

During the development of our intervention, we in fact included those diagnoses under CMD as we are aware about the frequent comorbidity among those conditions. [14] It is important to remember that there is an urgent need to develop simple packages of mental health care that can be implemented in remote settings and humanitarian emergencies. The recent Mental Health Gap project, launched by the World Health Organisation, focuses on 9 selected mental health conditions of public health importance. We believe that this is the way forward in order to scale up mental health care in under serviced areas [15].

As our main measurement instrument was developed through a process of simplification and adaptation of the SF-36, we should be cautious on interpreting levels of improvement on functionality. Although we acknowledge that using standardised instruments would have helped us to interpret our results and compare them with other studies, our experience has shown that most of the standardised instruments in mental health can not be used in routine mental health services in poor resource settings due to their length and need for specialised personnel. If outcome assessments are to be implemented in parallel to the process of scaling up of mental health services by using non-specialised health workers, simplified instruments need to be developed.

The lack of control group also limits a clear understanding of aspects of the intervention package that are essential. Different packages of care should be compared in different contexts in order to better understand other possible models for integration of mental health care into primary care in the context of a humanitarian emergency.

It is possible that the interviewer could have tended to mark better scores for patients at follow up. This would have biased our estimates. We believe this is unlikely as there was quality control by the supervisor psychiatrist.

Lastly, by having a sampling framework consisting of the first 6 months consecutive patients that attended mental health consultations, our results might only reflect the patients with higher chances of achieving good outcomes as they were able to reach our health facility and return to follow up consultations. Therefore, our positive results can not be generalised to the whole population with mental disorders in Habilla.

As we were unable to reach patients who were lost to follow up, the fact that they were not introduced in the analysis of outcomes remains as an important limitation of our study. In order to better understand bias due to loss to follow up, we have reanalysed the data by giving the defaulters the same disability scores as their last consultation. Although there was a reduction on the size of the mean differences, the results remained positive.

Conducting research in humanitarian emergencies poses numerous challenges. First, there are security risks to the staff and the possibility of rapid evacuation due to deterioration of the security situation can largely undermine the continuation of the medical activities. Second, the constant supply of psychotropic medication can be also interrupted in case of lack of continuous access to the implementation sites. This has to be strongly considered in advance and stocks of medication have to be kept at health centre level. Thirdly, the simplification and adaptation of diagnostic tools, treatment guidelines, training protocols and measurement instruments to be used need the support of experienced staff, which is not always available and willing to work in extremely violent contexts.

## Conclusion

Our study demonstrates that the integration of mental health into primary care in a humanitarian emergency is feasible. Patients with severe mental illness and epilepsy appear to benefit the most from mental health services at the beginning of the integration process. Positive outcomes should stimulate us to scale up those services in different settings. Simplification of measurement instruments and strategies to prevent defaulters need to be developed.

## Competing interests

The authors declare that they have no competing interests.

## Authors' contributions

RS had full access to all of the data in the study and takes responsibility for the integrity of the data and the accuracy of the data analysis. SY and SC participated in the study concept and design. RS performed the statistical analysis and interpretation of the data. RS, SY and SC participated in the critical revision of the manuscript. All authors read and approved the final manuscript.
